# Salmagundi

**Published:** 1884-05

**Authors:** 


### Salmagundi.

EDITED BY E. G. BETTY, D.D.S.

"A THOROUGH scholar carries a key with which to open every door in the mansion of knowledge."

CORRESPONDENTS will please not forget that communications to "Salmagundi" will always reach us "care Messrs. Spencer & Crocker."

"Oh, Professor," exclaimed sentimental old Mrs. Fishwacker, during a private organ recital in her new music room, "*do* pull out that sweet nux vomica stop once more."

DR. KEMPTON says that he applies iodoform to exposed pulps in this way: Heat a bit of gutta percha quite warm, dip it into the dry powder, and apply with the adhering powder next the pulp.

FERRY-BOAT AMENITIES.—A Chinaman came into the ladies' cabin on the ferry-boat and took a seat beside an Irish market woman. He seemed to want to make himself agreeable, and, rubbing his hands, remarked: "Belly cold." The woman looked at him with an air of contempt, and replied: "If you'd put your shirt inside your pants your belly wouldn't be cowld, you haythen blaggard."—*Exchange.*

"THE exclusive study of art will not make the best artist; the sole devotion of a lifetime to business will not make the best merchant; the acquirement of technical skill alone will not make the best mechanic. Knowledge of other things, mental drill in other branches, breadth of view, and power of sympathy will all tell upon the specific work in hand and raise it to a higher level than that of any mere specialist."

A LITERARY paper credits Ruskin with saying that "We want one man to be always thinking, and another to be always working, and we call one a gentleman and the other an operator; whereas the workman ought often to be thinking and the thinker often to be working, and both should be gentlemen in the best sense. The mass of society is made up of morbid thinkers and miserable workers. It is only by labor that thought can be made healthy, and only by thought that labor can be made happy, and the two cannot be separated with impunity."

---

DR. ERIC SATTLER, in a well-written paper upon "The Present Status of the Tubercle-Bacillus Question," recently read before the Cincinnati Medical Society, makes the following remark, which dentists know is only too true with regard to themselves and the bug theory of dental caries: "There is, perhaps, no subject in the whole range of medicine more grossly misinterpreted and less understood by the great mass of our profession than that now under consideration. Whether through ignorance of the real question under discussion, whether through preconceived notions and narrow trains of thought, there exists such a jumble of opinions, such a mass of misinterpreted facts, that it is difficult to extricate oneself from the meshes of this medical maze and assume a position which shall neither encroach too much on the favorite ground of the extreme enthusiasts on the one side, nor enter too far the sacred precincts of the germ iconoclasts on the other."

---

POWDERED blood cooked and dried has been given to lambs in addition to their ordinary vegetable diet, and they became exceedingly fat and well developed; while others, confined to vegetables alone during the same period, lost flesh considerably. It was suggested that this mode of alimentation be employed for children deprived of nurse, and a case cited of a child eighteen months old, who was attacked with rickets, and upon whom the treatment showed excellent results. This element or course of dietetics has never, to our knowledge, been suggested by any one in the dental profession, and while the subject of aliments and complementary foods is just now so prominent, why not give it a trial? Large quantities of fine, healthy beef blood are annually turned into the sewers from our *abattoirs*, some of which might in this way be of incalculable benefit to hundreds requiring for their osseous and dental systems the phosphates contained in it. Have we forgotten that the drinking of warm blood from the recently slaughtered animal was, not long ago, fashionable for the treatment of phthisis and anæmia?

---

TRIAL PLATES FOR THE LOWER JAW.--These often give us a vast deal of vexation, because, if not made stiff, they bend and displace the teeth they carry, much to our annoyance and loss of time. To obviate all these troubles, Dr. Samuel Wardle recommends that the plate be made of modeling composition (medium grade) and strengthened with *twisted* wire. Ordinary iron wire is annealed and two strands twisted together, the result being a wire that looks like a thin screw. This is cut into lengths of about three inches and kept on hand for ready use. Two pieces are generally required to stiffen a plate. Bend them to suit the form of the plate, warm in the spirit flame or gas jet, and press them into place in the composition. When cold, the plate is as stiff as thick lead; made in one-third the time and without injury to the model. Try it.

---

LOUISVILLE, KY., March 29, 1884.

EDITOR SALMAGUNDI: Can any dentist suggest a remedy for persistent nausea from wearing an upper denture? The patient is a lady, and has been so troubled for years, and has worn rubber plates of various shapes without relief. I have just completed a piece for her on the new "Rose Pearl," hoping that by working it on metal the smooth surface would be beneficial as well as the action of the camphor in it. The nausea is never felt when the mouth is at rest, but comes on suddenly while talking or eating, and is often very embarrassing. The sensitive point is confined to a line just within the palatal edge of the ridge all around, and can be excited by the touch of the finger. Patient suffers some from nasal catarrh, and the affections seem to be in some degree associated together, growing better or worse at the same times.

H. B. Tileston, D.D.S.

We have a case in which the nausea comes on while eating only, and as the plate is partial the patient removes it at meal time. In Dr. Tileston's case there is accompanying nasal catarrh, and under the circumstances we would recommend treatment of the nasal trouble and its cure, if possible, though we would like to hear from some one else upon the subject.

---

We have made some very serviceable artificial crowns on bicuspid roots in the following manner: Trim up the end of the root neatly, and drill a tolerably good sized hole into it in a direct line. Into this, carefully but firmly, introduce an ordinary steel screw, such as can be procured at the hardware store. Fit around the root a cage or cell of annealed, thin saw blade such as we use for other purposes, and of such a length as to come to a line with the required height of the crown. When adjusted, tie it with fine wire; or, if there is not sufficient space between the teeth, secure with waxed silk floss. Then mix a sufficient quantity of amalgam *very dry* and *mallet* it into the cage and around the screw with tolerably warm instruments. The patient may then be dismissed for two or three or four hours, with the cage *in situ*, keeping a thin disc of cork between some of the teeth to prevent contact for the time being. Upon returning, the cage is removed, and a few minutes with the file, corundum stone, and emery cloth make a good finish. The operation is speedily performed, is inexpensive, and makes a good masticator. The *dry* amalgam contains less mercury, and when malleted with warm instruments becomes hard in an hour or two, sometimes a little longer. The rubber dam may be used if there is sufficient projection of the root; but we do not think it necessary, the napkin keeping things dry long enough to pack in the amalgam.

---

The Therapeutic Application of Nitrous Oxide Gas.—From a series of experiments made in Prof. Botkin's laboratory in St. Petersburg, Dr. S. Klikowitsch (*Virchow's Archiv.* xliv., 2) draws the following conclusions:

1. Nitrous oxide gas is incapable of supporting respiration in animals and plants, and, like other indifferent gases, leads to death from asphyxia. The asphyxia produced by this gas, however, presents points of contrast to the asphyxia produced by other means.

2. Nitrous oxide gas produces no chemical or morphological changes in the blood of animals, but is dissolved in it and again eliminated, according to physical laws, without apparently being broken up into nitrogen and oxygen.

3. Anæsthesia with laughing gas is so closely associated with insufficient oxidation of the blood that it cannot be regarded as absolutely without danger, especially in diseases of the heart, lungs or blood vessels.

4. The association of laughing-gas with twenty per cent. of oxygen completely removes the possibility of asphyxia, and produces a number of results capable of therapeutic application.

5. Under the influence of the mixture of laughing-gas and twenty per cent. oxygen, in the majority of healthy subjects, the heart's pulsations are increased, the pulse-wave diminished, and the respiratory movements decreased in number and increased in depth; these effects pass off in from three to five minutes.

6. In six cases of weak heart-action, the above gaseous mixture produced no unfavorable results; on the other hand, the pulse was decreased in frequency and increased in strength. These effects lasted from one to two hours.

7. In cases of disturbed respiratory innervation the mixture of laughing-gas and oxygen regulated the respiratory rhythm and rapidly removed the subjective and objective signs of deficient oxidation of the blood.

8. This gaseous mixture acts as a transient anæsthetic, and in angina pectoris causes a rapid removal of suffering.

9. It is to be preferred to chloroform as an anæsthetic in labor.

10. Vomiting and cough of reflex origin are arrested by a few inhalations of this mixture of gases.—*Phil. Med. Times.*

